# Diphtheria

**Published:** 1899-09-30

**Authors:** P. Watson Williams

**Affiliations:** Physician to Clifton College


### Hospital Clinics and Medical Progress.

### DIPHTHERIA.

By P. Watson Williams, M.D. Lond.,
Physician to Clifton College.

**Definition.**—Diphtheria is a specific infectious disease, due wholly or in part to the Klebs-Loeffler bacillus, and primarily a local infection, generally commencing in the fauces, more rarely in the larynx, nose, or genital mucous membrane, or on the surface of a wound. Being essentially a local infection the initial symptoms are local, and accordingly cases may be classified as pharyngeal, laryngeal, nasal, or wound diphtheria, &c.

The term diphtheria, derived from the Greek διφθέρα, a piece of leather, or membrane, was invented by Bretonneau, in 1821, to denote a certain kind of specific inflammation which was characterised by the formation of a false fibrous membrane or pellicle. He maintained that malignant angina and membranous croup were due to the same infective agent, and that the presence of a false membrane was essential for their identification. Virchow, on anatomical grounds, drew a distinction between "croupous inflammation," fibrinous exudation without necrosis of the surface epithelium, and "diphtheritic inflammation," fibrinous exudation not on the surface only, but involving the epithelium, which is shed, the terms "croupous" and "diphtheritic" being applied in this anatomical sense to such membranous inflammations of the mucous membrane wherever occurring.

But the difference between croupous and diphtheritic inflammation is now known to be merely one of degree, and the same infective micro-organisms may give rise to either. Moreover, several varieties of micro-organisms, and various chemical irritants, are known to cause inflammation with false membrane and superficial necrosis, anatomically identical with Virchow's definition of diphtheritic inflammation, while on the other hand the specific disease associated with the Klebs-Loeffler bacillus may be unattended with the presence of any membranous exudation at all.

It would be convenient, and avoid much confusion, if the term diphtheria could be employed in a clinical sense to include all affections characterised by the presence of false membranes, their pathological distinction being indicated by such terms as staphylo-coccal, streptococcal, mixed Loefflerial, pure Loefflerial, scarlatinal, traumatic, diphtheria, &c. But the word diphtheria has become definitely associated with the disease due to the Klebs-Loeffler bacillus, and the term must be reserved for all its clinical variations, whether the resulting local inflammation be attended with the formation of false membrane or not. Every inflammation due, partly or wholly, to the Klebs-Loeffler bacillus is diphtheria, though the mere presence of the specific bacillus in the throat does not constitute diphtheria, for it sometimes occurs in healthy throats, such a condition being exactly analogous to the presence of leptothrix spores or mycelium, streptococci, staphylococci, or even tubercle bacilli in a normal throat.

**Etiology.**—The influence of age is very pronounced, both as regards the susceptibility to attack and the mortality. Children between the ages of three and fifteen are especially liable to contract diphtheria when exposed to infection, and it is in these first 15 years of life that the great majority of deaths occur.

It is remarkable that infants at the breast seldom contract diphtheria, and some investigations by Schmid and Pflanz[1] seem to show that in new-born children, and especially in the offspring of a mother whose blood contains diphtheria antitoxins, there is provided a store of these antitoxins collected during intra-uterine life from the maternal blood, and that this is replenished from the mother's milk, so as to render them less susceptible, or even immune to diphtheria. This, of course, would give breast-fed infants a great advantage over those brought up by artificial means.

The influence of season on the number of fatal cases from diphtheria is fairly constant, and, according to W. H. Power,[2] October and November constitute "the well-known season of normal extra activity of diphtheria," and in early spring there is a decided, though less pronounced, tendency for diphtheria to prevail.

The influence of school attendance must be held to very largely account for the increase in the number of attacks in the spring and autumn. Dixey,[3] from an analysis of the mortality rates from diphtheria in London during ten years, 1887-1897, has shown that there is a factor distinct from ordinary seasonal conditions sufficiently powerful to induce a fairly regular fall in the number of cases of fatal diphtheria at two definite times of the year, these times corresponding with the usual holiday periods of schools. A striking instance of the influence of school attendance in the spread of diphtheria is recorded by Louis Parkes.[4] It was in June and July, 1896, when of 50 cases of children of school age, 20 were among the girls and infants attending one of the Board schools; the boys, who had a separate playground, with an entrance in another street, remaining exempt.

Coincident with sanitary progress is the development of education amongst the masses, and there is ample reason to attribute the increased incidence of diphtheria in a large measure to the more general and regular attendance at day and Sunday schools.

The influence of sewer gas and insanitary environment is a moot question, but facts do not support the still very prevalent opinion that bad sanitation is directly a common cause of diphtheria, although it may have some indirect influence. Improved sanitation, which has characterised the last quarter of the nineteenth century, has been followed by a remarkable diminution of diseases that are known to depend on defective sanitation, viz., typhoid and typhus fevers; but during the same period there has been a progressive increase in the incidence of diphtheria, especially in towns where the progress in sanitation has been most marked.

Thorne Thorne[5] points out that "the quarter of a century beginning about the year 1870 has been a period in which unexampled progress has been made in England and Wales in improving the sanitary circumstances under which the people are living, whether of water supply, sewerage and drainage, the disposal of refuse and excreta, or dwelling accommodation. It has also been a period in which the rate of mortality from enteric fever—a disease known to be intimately associated with bad sanitary circumstances affecting water supply, sewerage, and drainage, and so forth—has fallen from 0·37 to 0·17 per thousand. But during the same period of sanitary progress the corresponding death-rate from diphtheria has gone up from 0·12 to 0·19, an increase of 63 per cent., and in London and the large cities the increase has been even greater—it has, indeed, more than trebled. . . . It is certain that in the vast majority of cases diphtheria is due either to infection by an antecedent case, whether in school or elsewhere, or to infection conveyed through milk; and that, consequently, the balance attributable to faulty sanitary circumstances cannot be a large one."

Shattock[6] has experimentally investigated the action of sewer gas in raising the toxicity of non-virulent diphtheria bacilli, for although diphtheria bacilli are not disengaged by evaporation from a fluid in which they are growing, and have never been isolated from sewer gas, it is conceivable that a non-virulent diphtheria bacillus, such as is present in the throat of many persons, might, in consequence of the inhalation of sewer-air, regain its virulence, and become the cause of a diphtheritic lesion, which, except for this factor, would not have arisen. One bacillus was subjected to the influence of a specially improvised badly-drained sewer for two months, and the other for five weeks, but in each case the bacilli were found on injection to have remained non-virulent.

Nevertheless the influence of sewer-gas may operate by causing sore throats, and thus rendering those exposed more susceptible to the infection by the diphtheria bacillus.

The nature of the soil has not been proved to have any direct influence in the occurrence of diphtheria, but it appears to be more prevalent in the eastern counties than in the west and south of England. Thorne Thorne[7] states that there is some ground for believing "that areas which favour retention in the soil of wetness and of dead organic matter, and are exposed to the influence of cold, wet winds, do tend to the fostering and fatality of diphtheria."

**Conveyance of Infection.**—Undoubtedly the infection is generally by aerial convection, either directly from expectorated particles from an infected individual, or from fomites, books, or playthings that have been exposed to contamination, or by direct inoculation by kissing, &c. A number of epidemics have been definitely traced to milk. But there is no doubt that infection may be carried by apparently healthy persons, numerous instances of this having been observed and recorded; for example, nurses and others who have been exposed to infection may carry the specific bacillus in their throats and, without showing any symptoms of the disease themselves, may communicate diphtheria in a virulent form to others.

Another often obscure source of infection is the existence of diphtheritic fibrinous rhinitis, and instances have occurred where a person in apparent health, but in whom fibrinous rhinitis has been found, have afforded the only possible source of a virulent attack of faucial diphtheria in those coming into close relations with them.

It seems hardly necessary to state that the Klebs-Loeffler bacillus may be found in the throat of an apparently healthy individual under one other set of circumstances, viz., when a person has had an attack of diphtheria, and in whom the bacillus persists in the throat or nose for a more or less considerable period after convalescence has been established.

Cats have been shown to suffer from diphtheria, and may then infect human beings. The diphtheria bacillus has been found by Klein in the patches of broncho-pneumonia in cats suffering from diphtheria; false membrane does not appear to be formed in the cat, the disease mainly affecting the lung, and causing also kidney affections and ophthalmia. The only other domestic animal known to become affected with diphtheria is the cow, in which the disease is known as "chapped teats."

The false diphtheria-like membranes sometimes found in pigeons and turkeys are due to organisms, but the Klebs-Loeffler bacillus has not been identified in connection with them.

**Prophylaxis.** — Inasmuch as any inflammatory or diseased condition of the mucous membrane of the upper respiratory tract renders an individual more susceptible of infection by the diphtheria bacillus, it is desirable to remove susceptible individuals from damp or insanitary surroundings, and combat any existing disease in the nose and throat by appropriate measures. Especially should chronically enlarged or hypertrophied tonsils be attended to.

During an epidemic of diphtheria all milk should be sterilised by boiling, or by heating to 140 deg. Fahr. for five minutes.

The importance of isolating patients who have recently had diphtheria, at any rate so long as diphtheria bacilli virulent to guinea-pigs can be detected by cultures taken from the throat or nose, is obvious, for no one can say that the bacilli, even if shown to be of slight virulence at the time, will not cause a severe or fatal attack in others who may be inoculated from them, and instances of this will be cited further on. Particularly is this rule important with regard to schools, for I cannot understand why it should not be considered necessary to be as careful in regard to diphtheria as in scarlatina. No practitioner would take the responsibility of permitting the mildest case of scarlatina or an ambulatory typhoid to mix with others in a school until absolutely free from infectiousness, and so long as Klebs-Loeffler bacilli persists in the throat of any child it ought to be regarded as potentially infectious and separated accordingly, at any rate until our means of determining the infective power of apparently non-virulent bacilli, morphologically indistinguishable from the virulent Klebs-Loeffler bacillus, is placed on a more satisfactory footing. If several cases of diphtheria break out in a school the question of closing the school must be carefully considered. If an elementary school it should undoubtedly be closed, but in the case of elder children, swabs should be taken from each individual, and if any are shown to have the bacillus they should be promptly isolated. Again, after a school has been closed on account of an outbreak no scholar should be allowed to return—at any rate, within three weeks—without a bacteriological examination of the throat, whether there is a previous history of diphtheria or not. Nor should any scholar be allowed to return to school from a house where diphtheria has occurred until at least twelve days have elapsed after all those at home have become convalescent, nor until he has himself been subjected to a bacteriological examination.

Post-scarlatinal diphtheria is especially prone to occur in hospitals for infectious diseases. Garratt and Washbourn[8] examined 666 cases of scarlet fever under their care at the London Fever Hospital, and in only 1·2 per cent. of the cases were found bacilli morphologically resembling the bacillus diphtheriæ. They succeeded in largely reducing the percentage of post-scarlatinal diphtherias by separating those in whom such bacilli were found.

Antitoxin serum injections have been given with considerable success for the purpose of producing immunity. In 1886 I collected reports of 12,488 cases in which these prophylactic injections had been given, and of these 61 cases subsequently contracted diphtheria. Two groups of cases may be cited, viz., 511 cases at the New York Infants' Asylum, reported by Peck, all being exposed to contagion, and quarantine measures having proved unavailing; no cases developed until three weeks had elapsed, then two contracted diphtheria; and 136 cases at the Nursery and Child's Hospital, reported by Allen Thomas; none of these cases contracted diphtheria, but a resident medical officer and a nurse, neither of whom were immunised, did. Klein and others have been able to render guinea-pigs immune by antitoxic serum injections, but complete immunity in guinea-pigs and in human beings appears not to extend beyond two or three weeks.

The value of protective inoculations is insufficiently recognised as yet, but it seems to have been strikingly exemplified during an epidemic at St. Johnsbury, Vt., reported by Jane Berry.[9] Two hundred and twenty-one children and young adults were inoculated with from 200 to 300 units, according to age, during the last week of November, 1897, and by the following January 16th not a single case of diphtheria had appeared among them after repeated subsequent exposure to contagion, though many cases had occurred in the schools during the interval.

[1] Wien. Klin. Woch., 1896, No. 42; Epit. Brit. Med. Jour., Oct. 9, 1897. [2] Cited by Thorne Thorne, Allbutt's System, vol. 1. [3] Brit. Med. Jour., Sept. 3, 1898. [4] Lancet, May 15, 1897. [5] Allbutt's System, vol. 1. [6] Lancet, Dec. 11, 1897. [7] Loc. cit. [8] Brit. Med.. Jour., April 15, 1899. [9] New York Med. Rec., Feb. 12, 1898.

*(To be continued.)*

